# Pyrophosphate-Dependent ATP Formation from Acetyl Coenzyme A in *Syntrophus aciditrophicus*, a New Twist on ATP Formation

**DOI:** 10.1128/mBio.01208-16

**Published:** 2016-08-16

**Authors:** Kimberly L. James, Luis A. Ríos-Hernández, Neil Q. Wofford, Housna Mouttaki, Jessica R. Sieber, Cody S. Sheik, Hong H. Nguyen, Yanan Yang, Yongming Xie, Jonathan Erde, Lars Rohlin, Elizabeth A. Karr, Joseph A. Loo, Rachel R. Ogorzalek Loo, Gregory B. Hurst, Robert P. Gunsalus, Luke I. Szweda, Michael J. McInerney

**Affiliations:** a Department of Microbiology and Plant Biology, University of Oklahoma, Norman, Oklahoma, USA; b Department of Chemistry and Biochemistry, University of California Los Angeles, Los Angeles, California, USA; c Department of Biological Chemistry, University of California Los Angeles, Los Angeles, California, USA; d Chemical Sciences Division, Oak Ridge National Laboratory, Oak Ridge, Tennessee, USA; e Department of Microbiology, Immunology and Molecular Genetics, University of California Los Angeles, Los Angeles, California, USA; f Aging and Metabolism Research Program, Oklahoma Medical Research Foundation, Oklahoma City, Oklahoma, USA

## Abstract

*Syntrophus aciditrophicus* is a model syntrophic bacterium that degrades key intermediates in anaerobic decomposition, such as benzoate, cyclohexane-1-carboxylate, and certain fatty acids, to acetate when grown with hydrogen-/formate-consuming microorganisms. ATP formation coupled to acetate production is the main source for energy conservation by *S. aciditrophicus*. However, the absence of homologs for phosphate acetyltransferase and acetate kinase in the genome of *S. aciditrophicus* leaves it unclear as to how ATP is formed, as most fermentative bacteria rely on these two enzymes to synthesize ATP from acetyl coenzyme A (CoA) and phosphate. Here, we combine transcriptomic, proteomic, metabolite, and enzymatic approaches to show that *S. aciditrophicus* uses AMP-forming, acetyl-CoA synthetase (Acs1) for ATP synthesis from acetyl-CoA. *acs1* mRNA and Acs1 were abundant in transcriptomes and proteomes, respectively, of *S. aciditrophicus* grown in pure culture and coculture. Cell extracts of *S. aciditrophicus* had low or undetectable acetate kinase and phosphate acetyltransferase activities but had high acetyl-CoA synthetase activity under all growth conditions tested. Both Acs1 purified from *S. aciditrophicus* and recombinantly produced Acs1 catalyzed ATP and acetate formation from acetyl-CoA, AMP, and pyrophosphate. High pyrophosphate levels and a high AMP-to-ATP ratio (5.9 ± 1.4) in *S. aciditrophicus* cells support the operation of Acs1 in the acetate-forming direction. Thus, *S. aciditrophicus* has a unique approach to conserve energy involving pyrophosphate, AMP, acetyl-CoA, and an AMP-forming, acetyl-CoA synthetase.

## IMPORTANCE

Bacteria use two enzymes, phosphate acetyltransferase and acetate kinase, to make ATP from acetyl-CoA, while acetate-forming archaea use a single enzyme, an ADP-forming, acetyl-CoA synthetase, to synthesize ATP and acetate from acetyl-CoA. *Syntrophus aciditrophicus* apparently relies on a different approach to conserve energy during acetyl-CoA metabolism, as its genome does not have homologs to the genes for phosphate acetyltransferase and acetate kinase. Here, we show that *S. aciditrophicus* uses an alternative approach, an AMP-forming, acetyl-CoA synthetase, to make ATP from acetyl-CoA. AMP-forming, acetyl-CoA synthetases were previously thought to function only in the activation of acetate to acetyl-CoA.

## INTRODUCTION

In anaerobic ecosystems, fatty and aromatic acid degradation is essential for complete conversion of cellulosic biomass to methane (1). *Syntrophus aciditrophicus* is a metabolic specialist capable of degrading short-chain fatty and aromatic acids (i.e., butyrate, benzoate, or cyclohexane-1-carboxylate) to acetate, CO_2_, formate, and H_2_ (2). Uniquely, for this metabolism to proceed, a hydrogenotrophic partner is necessary to remove hydrogen and formate so that catabolic reactions remain thermodynamically favorable (1). This cooperative metabolism, commonly referred to as syntrophy, operates near thermodynamic equilibrium (3); thus, syntrophic metabolizers such as *S. aciditrophicus* must utilize energy conservation mechanisms that operate at low Gibbs free energy changes.

The formation of ATP and acetate from acetyl coenzyme A (CoA), which is formed during the degradation of its substrates (benzoate, cyclohexane-1-carboxylate, and fatty acids), is an important source of ATP in *S. aciditrophicus* (2, 4). However, the mechanism used for ATP formation is unclear. Typically, acetogenic bacteria utilize two enzymes, phosphate acetyltransferase and acetate kinase, to synthesize ATP and acetate from acetyl-CoA (see Fig. S1 in the supplemental material). Yet, the genome of *S. aciditrophicus* lacks an acetate kinase homolog (4), and the enzymatic activities of acetate kinase and phosphate acetyltransferase were extremely low in cell extracts of *S. aciditrophicus*, *Syntrophus gentianae*, and *Syntrophus buswellii* (5, 6). Thus, an alternative enzymatic pathway for substrate-level phosphorylation must be present in *S. aciditrophicus.*

Acetate-producing archaea and some anaerobic eukaryotes synthesize ATP using ADP-forming, acetyl-CoA synthetases where ATP is synthesized in one step from acetyl-CoA, ADP, and phosphate (7, 8). The genome of *S. aciditrophicus* contains nine genes annotated as archaeal-type ADP-forming, acetyl-CoA synthetases (4) (see Fig. S1 in the supplemental material). *S. aciditrophicus* has two gene clusters, each with a butyrate kinase gene and two phosphate acetyl-/butyryltransferase genes that could function in the place of acetate kinase and phosphate acetyltransferase. Last, genetic and biochemical analyses suggested that *Aspergillus nidulans* synthesizes ATP from acetyl-CoA using an AMP-forming, acetyl-CoA synthetase, which synthesizes ATP from acetyl-CoA, AMP, and pyrophosphate (9). *S. aciditrophicus* contains two such AMP-forming, acetyl-CoA synthetase genes. The above genomic analysis reveals that *S. aciditrophicus* has the potential to utilize an ATP synthesis mechanism previously not described in bacteria.

Utilizing a multifaceted approach, which included shotgun transcriptomic and proteomic sequencing and metabolite and enzymatic analyses, we show that *S. aciditrophicus* generates ATP from substrate-level phosphorylation via pyrophosphate and the AMP-forming, acetyl-CoA synthetase. This mechanism for acetate and ATP production has yet to be described in bacteria but represents a novel energy conservation mechanism that may be present in other bacteria that recycle pyrophosphate generated from substrate activation or core molecule biosynthesis.

## RESULTS

### AMP-forming, acetyl-CoA synthetase is expressed and translated under all growth conditions.

**FIG 1:**
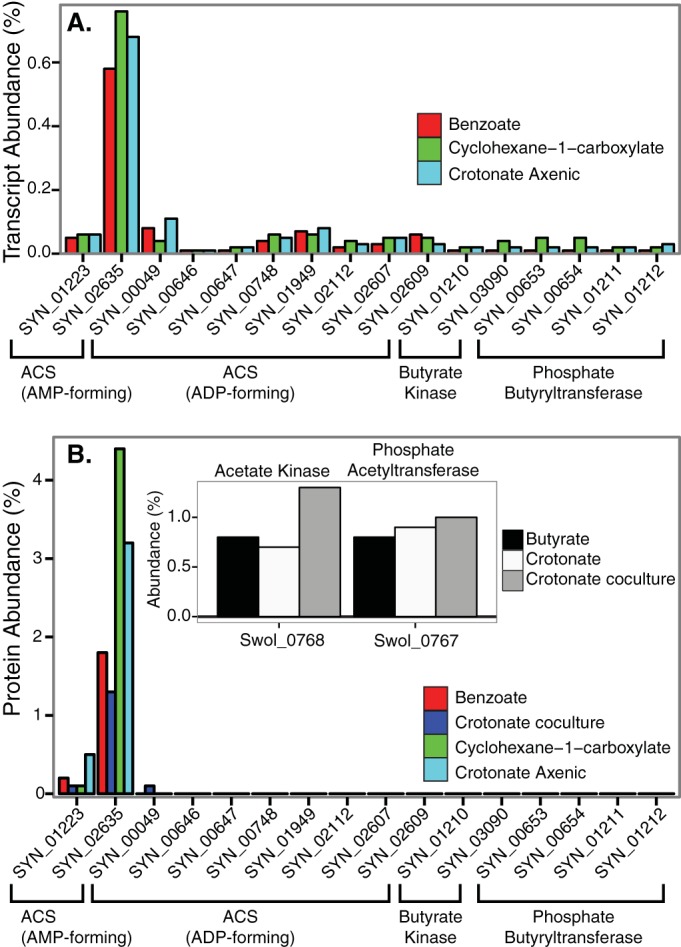
Abundance of transcripts and peptides of potential candidates for ATP synthesis by substrate-level phosphorylation in *S. aciditrophicus* grown under different conditions. (A) Transcript abundance in percentage of total RNA sequences detected in *S. aciditrophicus* grown in coculture with *M. hungatei* on benzoate, cyclohexane-1-carboxylate, and crotonate. (B) Peptide abundance in percentage of total peptide sequences detected in *S. aciditrophicus* grown in coculture with *M. hungatei* on benzoate, crotonate, and cyclohexane-1-carboxylate and in pure culture on crotonate. (Inset) Peptide abundance in percentage of total peptide sequences detected of annotated phosphate acetyltransferase and acetate kinase gene products in *S. wolfei* grown on crotonate and butyrate. The accession number for each gene is listed in parentheses after the gene locus tag number as follows: phosphate acetyl-/butyryltransferases, SYN_00653 (WP_011417962.1), SYN_00654 (WP_011417963.1), SYN_01211 (WP_011418341.1), and SYN_01212 (WP_011418342.1); acetate/butyrate kinase, SYN_03090 (WP_011417964.1) and SYN_01210 (WP_011418340.1); acetyl-CoA synthetase (ADP forming), SYN_00049 (WP_011418090.1), SYN_00646 (WP_011417955.1), SYN_00647 (WP_011417956.1), SYN_01949 (WP_011416704.1), SYN_02607 (WP_011416366.1), SYN_02609 (WP_011416368.1), SYN_00748 (WP_011418068.1), SYN_2112 (WP_011416964.1), and SYN_02878 (WP_011417834.1); acetyl-CoA synthetase (AMP forming), SYN_02635 (WP_011418543.1) and SYN_01223 (WP_011418354.1)

Enzymes that catalyze important catabolic functions such as ATP synthesis are expected to be highly expressed at the mRNA level and abundant in the total protein pool. Shotgun transcriptomics of *S. aciditrophicus* grown in coculture with *Methanospirillum hungatei* on crotonate, benzoate, and cyclohexane-1-carboxylate revealed that SYN_02635 (*acs1*) was highly expressed under all three growth conditions, compared to other target genes, representing 0.58 to 0.76% of the total transcripts detected (Fig. 1A; see also Table S1 in the supplemental material). In contrast, transcripts of SYN_01223 (*acs2*), the nine ADP-forming, acetyl-CoA synthetases, the two butyrate kinases, and two phosphate acetyl-/butyryltransferases each accounted for less than 0.1% of the total transcripts detected (Fig. 1A). Shotgun proteomic analysis revealed an AMP-forming, acetyl-CoA synthetase (Acs1), encoded by SYN_02635 (*acs1*), as one of the most abundant proteins in the proteome of *S. aciditrophicus* under all growth conditions (Fig. 1B; see also Table S1). Acs1 comprised 1.3, 1.8, and 4.4% of the total peptides detected when *S. aciditrophicus* was grown in coculture with *M. hungatei* on crotonate, benzoate, and cyclohexane-1-carboxylate, respectively. Additionally, Acs1 represented 3.2% of the total peptides detected when *S. aciditrophicus* was grown in pure culture on crotonate (Fig. 1B). In contrast, the ADP-dependent, acetyl-CoA synthetase (Acd) peptides were low. The most abundant Acd, the SYN_00049 gene product, was found only in crotonate-grown, coculture cells and comprised 0.08% of the total peptides detected. The two butyrate kinase genes and phosphate acetyl-/butyryltransferase genes were very low in abundance in the proteome of *S. aciditrophicus* (<0.05% of the total peptides detected) (Fig. 1B). For comparison, acetate kinase and phosphate acetyltransferase were abundant in the proteome of *Syntrophomonas wolfei*, which is known to use these two enzymes for ATP synthesis from acetyl-CoA (10) (Fig. 1B, inset).

### High acetyl-CoA synthetase activity detected in cell extracts.

Cell extracts of *S. aciditrophicus* had high acetyl-CoA synthetase activity and low or undetectable activities of acetate kinase and phosphate acetyltransferase, regardless of growth condition (Table 1). Under some growth conditions, acetate kinase, butyrate kinase, or phosphate acetyltransferase activity was detected, but the activity of its required partner protein, phosphate acetyltransferase or either of the two kinases, was low (Table 1). In comparison, acetate kinase and phosphate acetyltransferase activities in cell extracts of *S. wolfei*, our enzymatic control, were high (Table 1). Axenic cultures of the syntrophic partner *M. hungatei* had little to no detectable activity of any of the above enzymes (Table 1).

**TABLE 1:** Enzyme activities in cell extracts of *S. aciditrophicus* grown in pure culture and in coculture with *M. hungatei* and of *S. wolfei* grown in pure culture on crotonate^c^

Culture	Growth substrate	Activity of enzyme^b^			
		Acetyl-CoA synthetase^a^	Acetate kinase	Butyrate kinase	Phosphate acetyltransferase
*S. aciditrophicus*	Crotonate	4.86 ± 0.35	0.25 ± 0.05	0.19 ± 0.03	0.02 ± 0.007
	Crotonate coculture	0.92 ± 0.34	0.07 ± 0.03	0.09 ± 0.01	0.18 ± 0.03
	Benzoate coculture	0.53 ± 0.01	0.01 ± 0.001	BDL	BDL
	Cyclohexane-1-carboxylate coculture	7.4 ± 0.3	0.01 ± 0.001	BDL	BDL
*M. hungatei*	H_2_ + CO_2_	BDL	0.004 ± 0.002	BDL	BDL
*S. wolfei*	Crotonate	ND	0.37 ± 0.1	0.06 ± 0.002	0.97 ± 0.2

a The assay could not distinguish between ADP-forming and AMP-forming acetyl-CoA synthetase activities due to the adenylate kinase activity in *S. aciditrophicus* cell extracts.

b Mean ± standard deviation from triplicate determinations in units per milligram of protein.

c Coculture cells of *S. aciditrophicus* and *M. hungatei* were separated from each other by Percoll density gradient centrifugation as indicated. Abbreviations: BDL, below detection limit; ND, not determined.

The specific rate of acetate production by intact *S. aciditrophicus* cells grown in pure culture on crotonate was 1.18 ± 0.2 µmol ⋅ min^−1^ ⋅ mg of protein^−1^. The specific activities of acetate kinase, butyrate kinase, and phosphate acetyltransferase were all significantly lower than the resting cell acetate production rate (*P* ≤ 0.0014 for all comparisons). The acetyl-CoA synthetase activity in the acetyl-CoA formation direction in cell extracts of *S. aciditrophicus* (Table 1) was significantly higher (*P* < 0.0001; Student’s *t* test) than the resting cell acetate production rate. The acetyl-CoA synthetase activity in the acetate and ATP formation direction in cell extracts of *S. aciditrophicus* (Table 2) was the same (*P* = 0.50; Student’s *t* test) as the resting cell acetate production rates. The above rate comparisons show that only the acetyl-CoA synthetase activity accounts for the rate of acetate production by *S. aciditrophicus*.

**TABLE 2:** Inhibition of adenylate kinase by Ap5A to determine nucleotide specificity of acetyl-CoA synthetase activity in cell extracts of *S. aciditrophicus*^b^

Activity	Substrate	Ap5A (mM)	Sp act^a^
Adenylate kinase	ADP	0	3.38 ± 0.16
		0.75	0.63
		1.5	0.02
ADP-forming acetyl-CoA synthetase	Acetyl-CoA, KP_i_, ADP	1.5	0.02 ± 0.003
AMP-forming acetyl-CoA synthetase	AMP, PP_i_, plus above	1.5	1.3 ± 0.2

a Mean ± standard deviation from triplicate determinations in units per milligram of protein.

b Abbreviations: P_i_, phosphate; PP_i_, pyrophosphate.

### Adenylate specificity of the acetyl-CoA synthetase activity.

**FIG 2:**
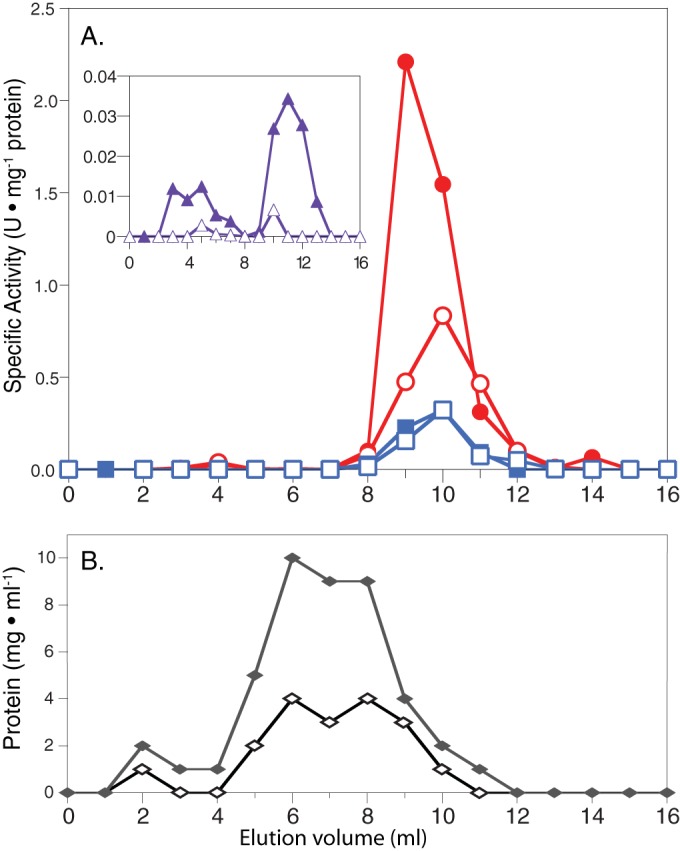
Gel filtration chromatography of the acetyl-CoA synthetase activity from cell extracts of *S. aciditrophicus* grown in pure culture on crotonate. There were two independent cultures (run 1 and run 2). (A) Specific activities of acetyl-CoA synthetases (units per microgram of protein): circles (closed, run 1; open, run 2), AMP-forming, acetyl-CoA synthetase in the acetyl-CoA-forming direction; squares (closed, run 1; open, run 2), AMP-forming, acetyl-CoA synthetase in the acetate-forming direction. (Inset) Specific activities of ADP-dependent, acetyl-CoA synthetase in the acetyl-CoA-forming direction (units per microgram of protein): triangles (closed, run 1; open, run 2). (B) Protein concentration: diamonds (closed, run 1; open, run 2). The amount of acetyl-CoA synthetase activity loaded on the column was 4.2 and 6.8 U, and the total amount of acetyl-CoA synthetase activity after chromatography was 4.8 and 5.6 U for run 1 and run 2, respectively.

The enzyme assay used to measure acetyl-CoA synthetase activity in cell extracts could not distinguish whether the activity was ADP forming or AMP forming because *S. aciditrophicus* cell extracts had adenylate kinase activity, which interconverts AMP to ADP in the presence of ATP*.* Two approaches were used to determine the nucleotide specificity of acetyl-CoA synthetase activity. First, an inhibitor of adenylate kinase, P1,P5-di(adenosine-5′)pentaphosphate (Ap5A), was added to cell extracts of crotonate-grown axenic *S. aciditrophicus* cells (11). The addition of 1.5 mM Ap5A inhibited 99% of the native adenylate kinase activity (Table 2). In the presence of Ap5A, no ADP-forming, acetyl-CoA synthetase activity was detected. Rather, high activity of an AMP- and pyrophosphate-dependent, acetyl-CoA synthetase was detected (Table 2). Second, we subjected duplicate cell extract preparations (run 1 and run 2) to size exclusion (Superdex) and gel filtration to separate acetyl-CoA synthetase and adenylate kinase activities (Fig. 2). The acetyl-CoA synthetase activity, measured in both the acetyl-CoA-forming and the acetate-forming directions, eluted prior to the adenylate kinase activity (Fig. 2). Adenylate kinase activity eluted as a single peak in fractions 10 to 15 (data not shown). An AMP- and pyrophosphate-dependent acetyl-CoA synthetase activity eluted from the column that accounted for 114 and 83% (for run 1 and run 2, respectively) of the acetyl-CoA synthetase added to the column (Fig. 2). ADP-forming, acetyl-CoA synthetase activity was detected but accounted for <2% of the total acetyl-CoA synthetase activity added to the column. Peptide analysis of the peak fraction of acetyl-CoA synthetase activity identified six unique peptides from *S. aciditrophicus* Acs1 (SYN_02635 gene product).

### Characterization of native and recombinant AMP-forming, acetyl-CoA synthetase.

Using ammonium sulfate fractionation, anion chromatography, hydroxyapatite chromatography, and affinity chromatography, the native acetyl-CoA synthetase activity in cell extracts of axenically grown *S. aciditrophicus* was purified to near homogeneity (75% yield; 94.8-fold purification) (see Table S2 in the supplemental material). Neither acetate kinase activity nor ADP-forming, acetyl-CoA synthetase activities were detected during the purification. The purified acetyl-CoA synthetase migrated as a single band on denaturing gel electrophoresis with a mass of ~74 kDa, consistent with the predicted mass for the SYN_02635 gene product. Peptide analysis identified the native acetyl-CoA synthetase as the SYN_02635 gene product (Acs1) based on matches to five unique peptides (4). The gene SYN_02635 was cloned and expressed in *Escherichia coli* BL21, and the histidine-tagged recombinant protein was purified and characterized (Table 3; see also Table S3 in the supplemental material). Both the purified native Acs1 and the recombinant Acs1 were specific for acetate as the substrate and had little activity with other tested organic acids, including known substrates that support growth of *S. aciditrophicus* (<0.6% of the acetate rate) (see Table S3). Both the purified native Acs1 and the recombinant SYN_02635 gene product used AMP and not ADP, and both were active in the acetate-forming and acetyl-CoA-forming directions (Table 3). The *K_m_*s for acetyl-CoA for the purified Acs1 and the recombinant Acs1 (0.41 ± 0.1 and 1.34 ± 0.5 mM, respectively) were within the known intracellular acetyl-CoA concentrations in microorganisms (0.2 to 1 mM) (12). The *K_m_* for pyrophosphate for the purified Acs1 was 0.25 ± 0.02 mM, consistent with the concentration (>0.2 mM) needed to operate in the ATP-forming direction.

**TABLE 3:** Kinetic parameters of the Acs1 purified from cell extracts of *S. aciditrophicus* and of recombinant Acs1 (SYN_02625 gene product) purified from cell extracts of *E. coli*

Enzyme	Reaction direction	Limiting substrate	*K_m_* (mM)^a^	*V*_max_^a^
Purified Acs1	Acetate→acetyl-CoA	Acetate	0.59 ± 0.2	74 ± 7
		CoA	0.26 ± 0.1	73 ± 16
	Acetyl-CoA→acetate	Acetyl-CoA	0.41 ± 0.1	7.5 ± 1.2
		Pyrophosphate^b^	0.25 ± 0.02	13.4 ± 0.35
Recombinant Acs1	Acetate→acetyl-CoA	Acetate	0.96 ± 0.02	89 ± 5.7
		CoA	0.21 ± 0.05	91 ± 7
	Acetyl-CoA→acetate	Acetyl-CoA	1.34 ± 0.5	1.2 ± 0.3

a Mean ± standard error.

b Acs1 was purified from cell extracts without ammonium sulfate fractionation.

### Intracellular concentrations of pyrophosphate and pyrophosphatase activity.

*S. aciditrophicus* cells maintained pools of pyrophosphate during growth (6.6 ± 0.8 nmol of pyrophosphate ⋅ mg of protein^−1^) twice as great as those maintained by *E. coli* (3.15 ± 0.2 nmol ⋅ mg of protein^−1^) (13).

The specific pyrophosphatase activity was 162.7 ± 7 nmol ⋅ mg of protein^−1^, of which 87% of the activity was found in the membrane fractions (141 ± 22 nmol ⋅ mg of protein^−1^). No pyrophosphatase activity was observed in the soluble fraction after ultracentrifugation of *S. aciditrophicus* extracts. These data are consistent with the genome of *S. aciditrophicus*, in which two genes for membrane-bound pyrophosphatases (SYN_02772 and SYN_02770) and no genes for soluble pyrophosphatases were detected (4).

### ATP, ADP, and AMP ratios.

*S. aciditrophicus* cells maintained elevated concentrations of AMP (2.1 ± 0.4 pmol of ATP ⋅ mg of protein^−1^) with an AMP-to-ATP ratio of 5.9 ± 1.4 during crotonate metabolism, which resulted in an energy charge of 0.24 ± 0.04 (Table 4). Almost all exponentially growing bacterial cells maintain an energy charge of >0.8 (14–16), which is what we measured for *E. coli* (0.93 ± 0.005).

**TABLE 4:** Adenylate levels and energy charge of *S. aciditrophicus* grown on crotonate medium and *E. coli* strain B grown on minimal glucose medium

Organism	Adenylate level^a^^,^^b^			Energy charge^b^
	ATP	ADP	AMP	
*S. aciditrophicus*	0.36 ± 0.07	0.89 ± 0.2	2.11 ± 0.4	0.24 ± 0.04
*E. coli* strain B	1.65 ± 0.4	0.28 ± 0.06	ND^c^	0.93 ± 0.005

a Picomoles per milligram of protein.

b Average values and standard deviations from three independent biological replicates and triplicate samples taken from each.

c ND, not detected.

## DISCUSSION

Acetate is a key metabolite that, depending on the biochemical process, can be taken up for cellular growth or biosynthesis and, in the case of acetate-producing microorganisms, can be excreted as a byproduct of metabolism. To date, bacteria have been shown to use only a two-step enzymatic reaction involving acetate kinase and phosphate acetyltransferase to convert acetyl-CoA to acetate and conserve energy in the form of ATP. However, for the bacterium *S. aciditrophicus*, genes for these enzymes are not present in its genome, and yet this organism’s catabolic machinery leads to the formation of acetate. Instead of the above classical bacterial route to make ATP from acetyl-CoA, *S. aciditrophicus* relies on an AMP-forming, acetyl-CoA synthetase for ATP synthesis from acetyl-CoA. AMP-forming, acetyl-CoA synthetases are thought to operate in the direction of acetyl-CoA formation; acetyl-CoA could then be used as a carbon source for biosynthesis or be oxidized to carbon dioxide for organisms that use acetate as the growth substrate (17, 18). The operation of the Acs reaction in the direction of acetyl-CoA formation is favored by the high ATP-to-AMP ratio (about 10) that bacteria normally maintain (14–16), However, the ATP-to-AMP ratio in *S. aciditrophicus* is very low (0.17 ± 0.04) (Table 4). The low ATP-to-AMP ratio and the high level of pyrophosphate would favor the operation of Acs1 in the direction of acetate formation.

For *S. aciditrophicus*, acetate is the primary carbon end product of its metabolism and acetyl-CoA is formed from its growth substrates (19). Thus, in *S. aciditrophicus*, Acs1 likely functions to form ATP from acetyl-CoA, AMP, and pyrophosphate rather than activating acetate to acetyl-CoA as AMP-forming, acetyl-CoA synthetases function in other bacteria. Acs1 is the only enzyme whose activity can account for the rate of acetate production by *S. aciditrophicus* (Table 1). Other enzymes potentially involved in substrate-level phosphorylation were in very low abundance in the proteome, and their activities were low or undetectable under the growth conditions tested (Fig. 1; Table 1). The only known instance of an AMP-forming, acetyl-CoA synthetase being used for ATP synthesis is when the fungus *A. nidulans* grows anaerobically on ethanol and nitrate (9).

**FIG 3:**
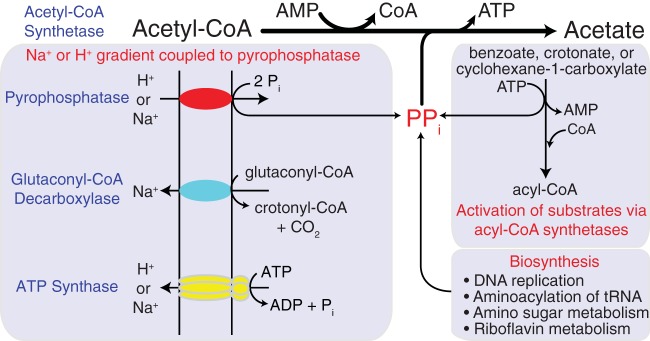
Importance of pyrophosphate cycling and Acs1 in the bioenergetics of *S. aciditrophicus*. Pyrophosphate formed during substrate activation or during biosynthesis is used to make ATP by Acs1. Additional pyrophosphate needed for the Acs1 reaction can be made by membrane-bound pyrophosphatases (red) using ion gradients formed by glutaconyl-CoA decarboxylase (blue) or ATP synthase (yellow).

The use of Acs1 by *S. aciditrophicus* for energy conservation provides another strategy for ATP synthesis in bacteria and suggests that pyrophosphate (PP_i_) is important in the energetics of *S. aciditrophicus* (Fig. 3). While PP_i_ is a byproduct of biosynthesis, a major source of pyrophosphate in *S. aciditrophicus* would be the activation of growth substrates such as benzoate, cyclohexane-1-carboxylate, and crotonate to their respective CoA intermediates by AMP-forming, acyl-CoA ligases (2), which results in pyrophosphate (PP_i_) formation (Fig. 3). The PP_i_ could then be used by Acs1 to form ATP from acetyl-CoA. Alternatively, PP_i_ could be hydrolyzed by membrane-bound pyrophosphatases (Fig. 3) whose activities were detected in membranes of *S. aciditrophicus*. Pyrophosphate hydrolysis by membrane-bound pyrophosphatases in *S. gentianae* is coupled to proton translocation (20); however, the stoichiometry of ATP-to-pyrophosphate hydrolysis was 1 ATP:3 PP_i_ (20). The formation of pyrophosphate during substrate activation and its use by Acs1 for ATP synthesis (Fig. 3) would result in a stoichiometry of 1 ATP:1 PP_i_ rather than a ratio of 1 ATP:3 PP_i_ if PP_i_ was used for the formation of a proton motive force by membrane-bound pyrophosphatases. Pyrophosphate cycling using Acs1 thus provides an efficient mechanism for energy conservation for *S. aciditrophicus*, which is critical for microorganisms that operate close to thermodynamic equilibrium (4). Many bacteria such as *S. wolfei* (10) use acetate CoA-transferases to activate fatty acids, which preserve the CoA thioester bond of acetyl-CoA by forming an acyl-CoA intermediate such as butyryl-CoA. Acetate CoA-transferases thus provide an energy-efficient mechanism for substrate activation, and pyrophosphate cycling would not be required.

It should be noted that the amount of PP_i_ formed from substrate activation is less than the amount of acetyl-CoA formed during substrate catabolism. For example, three acetyl-CoAs are formed during benzoate or cyclohexane carboxylate degradation, but only one PP_i_ is formed during benzoate or cyclohexane carboxylate activation. Additional PP_i_ needed during growth could be generated by membrane-bound pyrophosphates in *S. aciditrophicus* (Fig. 3). Proton-translocating enzymes such as the glutaconyl-CoA decarboxylase and/or ATP synthase can establish a proton motive force needed to generate additional PP_i_ via membrane-bound pyrophosphatases (Fig. 3). Cytoplasmic pyrophosphatase activity that would potentially hydrolyze PP_i_ was not detected in cell extracts of *S. aciditrophicus*. The lack of cytoplasmic pyrophosphatases allows *S. aciditrophicus* to maintain pyrophosphate levels twice as high as those found in *E. coli.*

Phylogenetic analysis among functionally characterized AMP-forming, acetyl-CoA synthetases reveals that *S. aciditrophicus* Acs1 forms a distinct clade with Acs proteins from other deltaproteobacteria, none of which are characterized biochemically or genetically. pBLAST analysis shows that Acs1 has the highest sequence similarity (73%) to an annotated peptide sequence from *Smithella* sp. strain ME-1, another syntrophic metabolizer (21). Amino acid alignments show that Acs1 contains the same active site residues and nucleotide-binding regions as found in other biochemically characterized Acs proteins (see Fig. S2 in the supplemental material). Further structure-function studies are needed to determine the structural features that may control the directionality of Acs1 in *S. aciditrophicus*.

Other bacteria may use an AMP-forming, acetyl-CoA synthetase for ATP synthesis. It is likely that *S. buswellii* and *S. gentianae* use the AMP-forming, acetyl-CoA synthetase for ATP synthesis, as both organisms have very low phosphate acetyltransferase and acetate kinase activities (5, 6). In addition, a survey of genomes of acetate-forming anaerobes revealed that several microorganisms lack genes for phosphate acetyltransferase, phosphate butyryltransferase, acetate kinase, or butyrate kinase (see Table S3 in the supplemental material). All of these microorganisms have a gene for an AMP-forming, acetyl-CoA synthetase (see Table S4).

Our work answers the critical question of how syntrophic bacteria such as *S. aciditrophicus* conserve energy for growth using catabolic reactions that operate close to thermodynamic equilibrium (3). The respiratory systems of syntrophic bacteria consume energy to drive the unfavorable redox reactions involved in hydrogen, formate, and reduced ferredoxin formation from high-potential electron donors such as NADH and reduced flavoproteins (22). Instead, syntrophic bacteria must rely on substrate-level phosphorylation reactions involving acetyl-CoA for ATP synthesis. Enzymatic analysis, metabolite profiling, and ^13^C-labeled acetate studies argue that the pathways for benzoate, cyclohexane-1-carboxylate and crotonate metabolism share a core set of enzymes that operate reversibly (2, 23), suggesting that these enzymes operate close to thermodynamic equilibrium. Purified and recombinant *S. aciditrophicus* Acs1 proteins are active in both the acetate-forming and acetyl-CoA forming directions as are other characterized Acs proteins (24). However, it is plausible and likely that Acs1’s primary role is to make ATP and acetyl-CoA, a function that has not been shown in bacteria, to our knowledge.

## MATERIALS AND METHODS

Detailed experimental procedures can be found in Text S1 in the supplemental material.

### Media and conditions for cultivation.

*Syntrophus aciditrophicus* strain SB (DSM 26646) and *Syntrophomonas wolfei* (DSM 2245B) (25) were grown in pure culture in medium containing crotonate. *S. aciditrophicus* was grown in coculture with *Methanospirillum hungatei* strain JF1 (ATCC 27890) on crotonate, benzoate, or cyclohexane-1-carboxylate. *S. wolfei* was grown in coculture with *M. hungatei* JF1 using crotonate or butyrate. *M. hungatei* strain JF1 was grown in pure culture on 80% H_2_-20% CO_2_ in the minimal medium supplemented with 5 mM acetate. *Escherichia coli* strain ATCC 11303 was grown on LB broth medium.

### RNA sequencing.

RNA was extracted using an RNeasy minikit (Qiagen, Mansfield, MA) and sent to the University of California—Los Angeles Genotyping and Sequencing Core Facility (Illumina, Inc., San Diego, CA, USA) for sample preparation, rRNA removal, cDNA synthesis, and Illumina sequencing. The SRA accession number for the RNAseq data is SRP079339.

### High-throughput proteome analysis.

Cells of *S. aciditrophicus* pure cultures and cocultures were sent to the University of California—Los Angeles for peptide analysis. Proteomic analyses of *S. wolfei* were performed as described previously (26). *S. aciditrophicus* proteomic data are available at the PRIDE proteomics data repository (http://www.ebi.ac.uk/pride/archive/) under accession number PXD004638.

### Cell harvesting, separation, and extract preparation.

Cultures were harvested by centrifugation, washed in anoxic phosphate buffer, and the pellets were stored at −80°C. *S. aciditrophicus* grown in coculture was separated from *M. hungatei* cells using a Percoll gradient as previously described (27). Cell extracts and membrane and soluble fractions were prepared as previously described (27).

### Enzyme assays.

All assays were performed at 37°C. A unit (U) of activity is defined as 1 µmol ⋅ min^−1^. Acetate kinase and butyrate kinase activities were measured in the direction of acid anhydride formation by following the formation of the hydroxymate (28). Phosphate acetyltransferase activity was measured in the direction of acetyl-CoA formation using arsenolysis combined with the hydroxymate assay as described previously (29, 30). AMP-forming Acs activity was measured in the direction of acetyl-CoA formation using a coupled enzyme assay where the oxidation of NAD was monitored (31). Adenylate kinase activity was measured using the same coupled enzyme assay without myokinase and using AMP and ATP as the substrate. ATP formation from acetyl-CoA, AMP, and pyrophosphate was measured by monitoring the reduction of NADP^+^ using hexokinase and glucose-6-phosphate dehydrogenase (32). This assay was used to determine the *K_m_* and *V*_max_ of the purified and recombinant Acs1. Pyrophosphatase activity was determined by measuring the loss of pyrophosphate colorimetrically by forming a phosphomolybdate complex that reacts in the presence of 2-mercaptoethanol (33).

### Resting cell acetate production rate.

Crotonate-grown *S. aciditrophicus* cells were harvested anaerobically in late log phase by centrifugation, and the cells were washed and resuspended in 50 mM anoxic phosphate buffer. Washed cells were amended with 10 mM crotonate, and samples for protein, crotonate, and acetate concentrations were taken with time.

### Inhibition of native adenylate kinase.

Adenylate kinase activity was inhibited with the addition of P1,P5-di(adenosine-5′)pentaphosphate (11). ADP, phosphate, and acetyl-CoA were added, and the absorbance was measured after the addition of each substrate to determine which components were needed for NADP^+^ reduction. Next, AMP and pyrophosphate were added to the reaction mixture, and activity was measured after each addition to verify the presence of an AMP-forming, acetyl-CoA synthetase activity.

### Gel filtration.

Cell extracts of *S. aciditrophicus* were loaded onto a Superdex 200 10/300 GL column (GE Healthcare), and fractions were assayed for Acs activity in the acetate-forming and acetyl-CoA-forming directions and for adenylate kinase activities. Fractions with high Acs activity were sent for peptide sequencing (University of Oklahoma Health Sciences Center Proteomics Core Facility, Oklahoma City, OK, USA).

### Purification of acetate-producing activity.

The dominant acetyl-CoA synthetase activity was purified from cell extracts obtained from crotonate-grown *S. aciditrophicus* cells using a combination of 45% ammonium sulfate fractionation, anion chromatography, hydroxyapatite chromatography, and affinity chromatography with a reactive green column. After reactive green chromatography, Acs migrated as a single band on sodium dodecyl sulfate gel electrophoresis. The band was excised and sent for peptide sequencing (University of Oklahoma Health Sciences Center Proteomics Core Facility, Oklahoma City, OK, USA). Kinetic constants were determined for the homogenous reactive green fraction by nonlinear regression data analysis.

### Expression of SYN_02635.

Gene SYN_02635, annotated as an AMP-forming, acetyl-CoA synthetase, was amplified, cloned and overexpressed using the Invitrogen PET_101 cloning system. The recombinant protein was purified by Ni-affinity chromatography. The *K_m_* was determined for the SYN_02635 gene product with acetate and CoA as described for the Acs purified from *S. aciditrophicus* cell extracts.

### Pyrophosphate measurement.

*S. aciditrophicus* was grown in pure culture on crotonate medium (50 ml) without resazurin and cysteine hydrochloride. Duplicate *S. aciditrophicus* cultures were immediately added to ice-cold 100% trichloroacetic acid (TCA) (1 volume of culture to 2 volumes of TCA) to stop metabolism and lyse the cells. After 10 min, the pH was adjusted to between pH 6 and 8 and pyrophosphate was measured colorimetrically as previously described (33).

### Adenylate measurements.

*S. aciditrophicus* was grown in pure culture on 20 mM crotonate medium in triplicate 200-ml volumes. Cultures were grown to mid-log phase, concentrated by centrifugation, resuspended in 10 ml of medium, and incubated for 24 h. After incubation, 1 ml of the culture was taken and immediately put into 500 µl of ice-cold 1 M KOH and subjected to bead beating using Lysing Matrix E (MP Biomedicals) for 1 min. Sample was centrifuged and stored at −80°C. High-performance liquid chromatography (HPLC) and a UV-visible (UV-Vis) diode array spectrometer were used to resolve and detect AMP and ATP as described previously (21). *E. coli* strain B was grown in 200 ml minimal glucose medium aerobically as a control. *E. coli* cultures were concentrated and harvested as described above, with the exception of the incubation being adjusted to 40 min after centrifugation.

### Homology searches.

BLASTn and BLASTp were used to identify nucleotide and amino acid sequences, respectively, homologous to SYN_02635 in the NCBI database. Homology amino acid sequence searches were also performed using the UniProtKB/Swiss-Prot (Swiss-Prot) database and enhanced domain database.

### Analytical procedures.

Substrates were measured by HPLC and acetate was measured by gas chromatography (27).

## SUPPLEMENTAL MATERIAL

Text S1  Supplemental materials and methods. Download Text S1, DOCX file, 0.2 MB

Figure S1  Potential mechanisms for ATP synthesis by substrate-level phosphorylation in *S. aciditrophicus*. Locus tag identification number is given for each gene. Download Figure S1, PDF file, 0.2 MB

Figure S2  Amino acid alignments of Acs1 and other representative acetyl-CoA synthetases whose biochemical function has been demonstrated. Blue denotes the known nucleotide binding sites, and green denotes the known active site amino acids. Download Figure S2, PDF file, 0.4 MB

Table S1  Transcript and proteomic data of housekeeping and substrate-level phosphorylation genes and proteins, respectively, in *Syntrophus aciditrophicus* Table S1, DOCX file, 0.1 MB

Table S2  Purification of the acetyl-CoA synthetase activity from cell extracts of *S. aciditrophicus* grown on crotonate. Table S2, DOCX file, 0.1 MB

Table S3  Substrate specificity of Acs1 purified from cell extracts of *S. aciditrophicus* grown on crotonate and for the purified recombinant Acs1 (SYN_02635 gene product). Table S3, DOCX file, 0.1 MB

Table S4  Members of the *Clostridia* that have a gene for an AMP-forming, acetyl-CoA synthetase (ACS) and lack either one or all of the following: acetate kinase (AK), butyrate kinase (BK), phosphate acetyltransacetylase (PTA), and phosphate butyryltransferase (PTB). Table S4, DOCX file, 0.1 MB
