# Supplementary material for: Pyrophosphate-Dependent ATP Formation from Acetyl Coenzyme A in Syntrophus aciditrophicus, a New Twist on ATP Formation
Source: mBio. 2016 Aug 16;7(4):e01208-16. doi: 10.1128/mBio.01208-16 (PMC4992975; doi:10.1128/mBio.01208-16)
Supplement: Text S1 — Supplemental materials and methods. Download [file mbo004162932s1.docx]

Supplemental Materials and Methods S1

**Media and conditions for cultivation.** *Syntrophus aciditrophicus* strain SB (DSM 26646) was grown anaerobically in a minimal medium without rumen fluid ([1](#_ENREF_1)). The Wolin's metal solution was modified to include Na_2_MoO_4_^,^2H_2_O (0.01g/L) Na_2_SeO_4_ (0.01g/L) and Na_2_WO_4_^.^2H_2_O (0.01g/L) ([2](#_ENREF_2)). Stock solutions of the modified Wolin's trace metals and Balch vitamins were added at a volume of 5 ml/L and 10 ml/L ([2](#_ENREF_2)). The headspace was pressurized to 27.5 kPa with a mixture of N_2_/CO_2_ (80%:20%, vol/vol). *S. aciditrophicus* (DSM 26646) was grown in pure culture on 20 mM crotonate and in coculture with *Methanospirillum hungatei* JF1 (ATCC 27890) on 20 mM crotonate, 10 mM benzoate, or 10 mM cyclohexane-1-carboxylate. *Syntrophomonas wolfei* (DSM 2245B) ([3](#_ENREF_3)) pure culture was grown in above minimal medium with 20 mM crotonate and cocultures of *S. wolfei* with *M. hungatei* strain JF1 were grown anaerobically in the above minimal medium with 20 mM crotonate or 20 mM butyrate. *M. hungatei* was grown in pure culture in 160-ml serum bottles in the above minimal medium with 5 mM acetate. Cultures of *M. hungatei* were pressurized daily with H_2_/CO_2_ (80% : 20%, vol/vol) to 137 kPa ([2](#_ENREF_2)). All cultures were checked for contamination by microscopic analysis and using thioglycollate medium, which does not support the growth of any of the above organisms. *Escherichia coli* strain ATCC 11303 was grown on Luria-Bertani (LB) broth medium. *E. coli* strain B was grown on minimal glucose medium ([4](#_ENREF_4)).

**RNA-sequencing.** *S. aciditrophicus* was grown in pure culture and coculture in triplicate 100-ml volume cultures until 50% substrate loss occurred and then each culture was transferred to sterile medium of the same composition using 30% inoculum. After the third consecutive transfer, cells were harvested at 50% substrate loss. Cell activity was slowed by quickly cooling to 4°C using dry ice and ethanol bath ([2](#_ENREF_2)). Cultures were then centrifuged (14,300 x g, 20 min, 4°C), and the cell pellets were resuspended in 1 ml of RNA later (Invitrogen). RNA was extracted using a Qiagen RNA extraction kit.

RNA was sent to University of California-Los Angeles Genotyping and Sequencing Core Facility (Illumina, Inc. 9885 Towne Centre Drive, San Diego, CA 92121 USA) for sample preparation, ribosomal removal, cDNA synthesis, and Illumina sequencing. Raw data files were received in FASTA format and processed as described previously ([5](#_ENREF_5), [6](#_ENREF_6)).

High-throughput proteome analysis. S. aciditrophicus was grown in pure culture and in coculture as described above in duplicate 500-ml Schott bottles with 250 ml of medium. Cell pellets were sent to University of California-Los Angeles for peptide analysis. Washed cell pellets were trypsin-digested in solution with deoxycholate present, in accordance with the eFASP method ([7-11](#_ENREF_7)). Tryptic digests were analyzed by shotgun LC-MS^E^ analysis or fractionated by hydrophilic interaction chromatography (HILIC) prior to LC-MS^E^ analysis. Peptides were separated by ultrahigh performance liquid chromatography on a Waters nanoacquity system equipped with a nanoelectrospray (nano-ESI) interface and a Waters Synapt quadrupole time-of-flight mass spectrometer. Accurate mass data for precursor and product ions were collected from 100-2000 m/z, with the data-independent acquisition alternating between low and elevated energy at 1-second intervals, for the duration of analysis. The lock mass channel was sampled every 30 seconds for calibration of MS and MS^E^ data. Raw data files were processed in ProteinLynx Global Server 2.5.2 (PLGS, Waters) to provide an inventory of precursor ions along with their respective fragment ions, to search against a combined database composed of the Uniprot HAMAP complete proteome for S. aciditrophicus (strain SB, August 2011) and the Uniprot HAMAP complete proteome for M. hungatei (strain JF-1 / DSM 864, August 2011), for peptide and protein identifications. Database search settings employed two missed cleavages, a peptide 4% false positive rate, fixed S-pyridylethyl modification of cysteine residues, and variable oxidation modification of methionine residues. The Waters Expression Informatics component of the PLGS software was utilized to quantify data using glycogen phosphorylase B (Uniprot accession P00489) as the quantification reference ([12](#_ENREF_12), [13](#_ENREF_13)). Tools in Data Extraction and Correction (DECO), a custom MS^E^ data-analysis software suite, were utilized for peptide-level quantitative and physiochemical comparative analyses ([7](#_ENREF_7)).

*S. wolfei* was grown in pure culture and coculture and the proteome was analyzed as described previously ([14](#_ENREF_14)).

**Cell harvesting and preparation of cell extracts.** For enzyme activity determination and enzyme purification, cultures were grown in triplicate 1.5-L volumes with the substrates indicated above, and harvested at mid-log phase by centrifugation (14,300 x g, 20min, 4°C). Cells were washed in 50 mM anoxic phosphate buffer (pH 8.5). Pellets were stored at -80°C until use. For cell-free extract preparation, pellets were broken by French press (82.7 MPa), and the lysate was centrifuged (14,300 x g, 20 min, 4°C), and designated the cell-free extract. Membrane and soluble fractions were prepared by ultracentrifugation of cell-free extracts (23). For coculture pellets, *S. aciditrophicus* cells were separated from *M. hungatei* cells using Percoll density gradient centrifugation as described previously ([2](#_ENREF_2)). Microscopic analysis showed the *S. aciditrophicus* fraction had less than 1% contamination by *M. hungatei* cells. For extract preparations from induced proteins in *E. coli*, cells were broken by French press (82.7 MPa) and the lysate was ultracentrifuged (23) to remove inclusion bodies and cell debris.

**Enzyme assays.** All enzyme assays were performed anaerobically and aerobically to determine if the activities were affected by oxygen. All activities were linear with time and proportional with the protein concentration. Controls for all assays included the deletion of each substrate and the cell-free extract, and the use of heat-treated extracts. All assays were performed at 37°C. Assay buffers were transferred in 1 cm cuvettes and warmed to 37°C in water bath prior to addition of reagents.

Acetate kinase and butyrate kinase activities were determined by following the formation of the hydroxymate ([15](#_ENREF_15)). The assay mixture contained 50 mM tris(hydroxymethyl)aminomethane (Tris) (pH 8.3), 10 mM ATP, 10 mM magnesium chloride, 0.5 M hydroxylamine, and 20 mM potassium acetate or sodium butyrate. The reaction was stopped after 20 minutes at 37°C by the addition of ferric reagent (10% iron chloride, 3% trichloroacetic acid, in 0.7 N hydrochloric acid) and the reaction mixture was centrifuged for 5 minutes at 13,000 x g. The molar extinction coefficient for acetyl hydroxamate was 594 M^-1^cm^-1^ at 535 nm.

Phosphate acetyltransferase activity was measured using arsenolysis combined with the hydroxymate assay as described previously ([16](#_ENREF_16), [17](#_ENREF_17)). The reaction mixture contained 10 mM Tris-HCl buffer (pH 8.6), 6 mM acetyl phosphate, 100 mM cysteine, 100 mM potassium chloride, and 500 µM CoA. The reaction was started with the addition of 50 mM sodium arsenate and incubated for 45 minutes at 37°C. The reaction mixture was then diluted 1:1 with 2 M hydroxylamine and neutralized with potassium hydroxide. After 5 minutes, the reaction was stopped by the addition of trichloroacetic acid to a final concentration of 5%. The reaction mixture was brought to a final volume of 1.5 ml with 2.5% iron chloride in 2 M hydrochloric acid and incubated for 15 minutes at room temperature. The mixture was then centrifuged for 5 minutes at 13,000 x g and the absorbance at 540 nm was determined. Acetyl-phosphate standard curve was generated, and used to determine the remaining acetyl-phosphate in assay mixture.

AMP-forming, acetyl-CoA synthetase activities were measured by coupling AMP formation with myokinase, pyruvate kinase (PK) and lactate dehydrogenase (LDH) to measure the oxidation of reduced nicotinamide adenine dinucleotide (NADH) spectrophotometrically at 340 nm ([18](#_ENREF_18)). The assay mixture contained 50 mM Tris-HCl buffer (pH 8.5), 10 mM magnesium chloride, 5 mM ATP, 1 mM phosphoenolpyruvate (PEP), 375 µM NADH, 2.8 U myokinase, 2.2 U pyruvate kinase, 2.2 U lactate dehydrogenase, and 480 µM coenzyme A (CoA). The reaction was started with the addition of the fatty acid. ADP-forming, acetyl-CoA synthetase activity was measured as above with the deletion of myokinase from the reaction mixture. Adenylate kinase activity was measured as above with AMP and no acetate and no myokinase added. The oxidation of NADH was measured at 340 nm and the extinction coefficient was 6220 M^-1^ cm^-1^ ([19](#_ENREF_19)).

The ability of the purified Acs1 and recombinant Acs1 to make ATP from acetyl-CoA, AMP, and pyrophosphate was determined by coupling ATP formation to the reduction of NADP^+^ at 340 nm using hexokinase and glucose-6-phosphate dehydrogenase ([20](#_ENREF_20)). The reaction mixture contained: 11.3 mM triethanolamine (TEA) (pH 7.8), 2.8 mM magnesium chloride, 1.1 mM NADP^+^, 5.6 mM glucose, and 9 µg of glucose-6-phosphate dehydrogenase/hexokinase  (Roche, Indianapolis, IN), pyrophosphate tetrasodium salt (0.5-2 mM), AMP (0.05-2 mM), and acetyl-CoA (0.02-2 mM). The reaction was started with the addition of acetyl-CoA or the enzyme. The extinction coefficient for NADPH is 6220 M-1 cm-1 ([19](#_ENREF_19)). This assay was also used to determine the K_m_ and V_max_ of the purified Acs1 and recombinant Acs1. One Unit (U) is equivalent to one µmol• min^-1^.

**Resting cell acetate production rate.** *S. aciditrophicus* was grown axenically on crotonate in 1.5-L volumes. The cells were harvested anaerobically in late log phase by centrifugation (14,300 x g for 20 minutes at 24°C). Cell pellets were washed three times by resuspending the pellet in 50 mM anoxic phosphate buffer reduced with 2.5% cysteine sulfide (pH 7.5) and followed by centrifugation as above. The final pellet was resuspended in 375 ml of resting medium (minimal media without ammonium chloride and vitamin solution) and incubated. After no increase in protein concentration was observed, the cells were washed and pelleted as described above. The cell pellet was resuspended in 10 ml of 50 mM anoxic phosphate buffer, and the suspension was transferred to serum bottles with 75 ml of resting medium with 1mM crotonate and incubated overnight at 37°C. To test if acetate concentrations were proportional with cell concentrations three inoculum volumes (2.5 ml, 0.5 ml, 0.025 ml) were used, each of which was done in triplicate. Bottles were then amended with 10 mM crotonate, and samples were taken before the addition of crotonate and every 30 minutes there after to measure protein, crotonate, and acetate concentrations. Incubations with cells and without crotonate, with crotonate and without cells, and without cells and without crotonate served as controls. All solutions and materials used to prepare and incubate resting cell suspensions were sterile.

**Inhibition of native adenylate kinase.** Adenylate kinase activity in cell-free extracts of *S. aciditrophicus* was inhibited with the addition of P1, P5-Di(Adenosine-5’)Pentaphosphate (Ap5A) ([21](#_ENREF_21)). Cell-free extracts were desalted on a G20 column and adenylate kinase activity was measured by coupling ATP formation from ADP with the reduction of NADP^+^ using glucose-6-phosphate dehydrogenase and hexokinase ([20](#_ENREF_20)). Ap5A was added in a range of 0 to 1.5 mM until adenylate kinase activity was inhibited. Then, ADP, phosphate, and acetyl-CoA were sequentially added to measure ADP-forming, acetyl-CoA synthetase activity. The absorbance at 340 nm was measured after each addition of substrate to determine which components were needed for NADP^+^ reduction. Next, AMP and pyrophosphate were sequentially added to the reaction mixture, and activity was measured after each addition to determine if an AMP-forming, acetyl-CoA synthetase activity was present.

**Separation of adenylate kinase and acetyl-CoA synthetase activities**. To determine the nucleotide specificity of acetyl-CoA synthetase, duplicate cell-free extract preparations of *S. aciditrophicus* were filtered with a 0.2 µm filter and 250 µl loaded onto a SuperdexTM 200 10/300 GL column (GE Healthcare). Proteins were eluted with 20 mM TEA and 100 mM NaCl (pH 8) buffer at a flow rate of 0.4 ml per minute. One-milliliter fractions were collected. Fractions 1 to 30 were assayed for acetate-forming and acetyl-CoA-forming activity both with ADP and AMP as substrates. Each fraction was also tested for adenylate kinase activity. Protein concentrations were measured for each fraction assayed and specific activities were determined. All fractions that had activities were assayed either without acetate/CoA or acetyl-CoA/pyrophosphate as controls. Fractions with peak activity were sent to the Molecular Biology and Cytometry Research (Oklahoma City, USA) peptide sequencing by high performance liquid chromatography and tandem mass spectrometry (HPLC/MS/MS) (University of Oklahoma Health Sciences Center Proteomics Core Facility, Oklahoma City, OK 73104).

**Purification of acetate-producing activity.** The dominant acetyl-CoA synthetase activity was purified from cell-free extracts prepared from approximately two grams of crotonate-grown, pure culture *S. aciditrophicus* cells. The cell-free extracts were treated with 45% ammonium sulfate and centrifuged (21,000 x g, 10 minutes, 4°C). The soluble portion was desalted on a G20 column equilibrated with diethylaminoethyl (DEAE) binding buffer (20 mM triethanolamine (TEA) and 5 mM magnesium chloride, pH 7.8). Three milliliters of the desalted ammonium sulfate fraction were loaded onto a DEAE column at a flow rate of 3 ml per minute. Proteins were eluted with DEAE elution buffer (DEAE binding buffer with 500 mM sodium chloride, pH 7.8) with a linear gradient of 0 to 200 mM sodium chloride and 1.5 ml fractions were collected. All fractions were assayed for both AMP-forming and ADP-forming acetyl-CoA synthetase activities. The active acetyl-CoA synthetase fractions were pooled, concentrated, and desalted using an Amicon filtration device with a 30-kDA molecular weight membrane filter (Millipore). The concentrated activity was loaded onto a hydroxyapatite column equilibrated with hydroxyapatite buffer (20 mM TEA, 5 mM magnesium chloride, and 5 mM potassium phosphate, pH 7.8) and the activity was eluted by using a step gradient with hydroxyapatite buffer with potassium phosphate concentrations of 5 mM, 20 mM, 40 mM, 60 mM, 100 mM, and 200 mM. The acetyl-CoA synthetase activity eluted at 40 mM potassium phosphate. Active fractions were pooled and concentrated with a 30-kDa molecular weight cut off membrane and washed with DEAE binding buffer. Two milliliters of concentrated active fraction were added to a reactive green column equilibrated with DEAE binding buffer. Proteins were eluted with DEAE elution buffer. Acetyl-CoA synthetase activity was eluted in 500 mM sodium chloride. The reactive green fraction was analyzed on sodium dodecyl sulfate gel electrophoresis (SDS-gel) and a single band was observed. The band was excised and sent to the laboratory for Molecular Biology and Cytometry Research (Oklahoma City, USA) for in gel trypsin digestion and sequencing (University of Oklahoma Health Sciences Center Proteomics Core Facility, Oklahoma City, OK 73104).

Kinetic constants were determined for the homogenous reactive green fraction by non-linear regression data analysis fit to the Michaelis-Menten equation on Kaleidagraph (Synergy Software, Reading, PA). The K_m_ for purified acetyl-CoA synthetase fraction from *S. aciditrophicus* was determined with the following range of substrates: acetate (0.05 - 6 mM) and CoA (0.03 - 0.6 mM) with the respective substrates kept at saturation. The K_m_ for acetyl-CoA was determined using a concentration range from 0.2 - 2 mM. A second purification of the acetyl-CoA synthetase activity was performed without ammonium sulfate fractionation to determine K_m_ for pyrophosphate (0.003 – 4 mM).

**Expression of SYN_02635.** Gene SYN_02635, annotated as an AMP-forming, acetyl-CoA synthetase, was amplified from *S. aciditrophicus* DNA using primers SYN_02635F, ^5'^CACCATGGGAGAAGAGTCGATAT^3'^, and SYN_02635R, ^5'^CAGTCTGTTCTTTACCAGATCGTCC^3'^. Amplification was done using Phusion DNA polymerase (Fermentas) with the following PCR parameters: 95°C for 5 min; 30 cycles of 95°C for 1 min, 60 °C for 1 min, 72°C for 1 min; and 72°C for 5 min. The PCR product was ligated into Invitrogen PET_101 vector and transformed into *E. coli* Top 10 cells. Plasmid constructs were sequenced by Oklahoma Memorial Research Foundation DNA Sequencing Facility. Plasmid 1074c had the correct nucleotide sequence and used for further expression into *E. coli* BL21. Protein expression was induced with 1 mM IPTG (isopropyl-β-d-thiogalactopyranoside) and incubated overnight at 18°C. The recombinant protein was purified by Ni-affinity chromatography and was eluted from the column with 250 mM imidazole. The K_m_ was determined for SYN_02635 gene product with the acetate range 0.4-10 mM, and CoA range 0.004 - 5 mM.

**Internal pyrophosphate measurements and pyrophosphatase activity.** *S. aciditrophicus* was grown in pure culture on 20 mM crotonate medium described above with the removal of both resazurin and cysteine hydrochloride. A needle attached to small-bore tubing was inserted through the stopper of each culture bottle and the culture was by pressurized with 55 kPa N_2_/CO_2_ (80%: 20%, vol/vol). The bottle was inverted and the culture fluid was pushed through a second needle attached to small-bore tubing into pre-weighed flasks containing 20 ml of rapidly stirred, ice cold 100% trichloroacetic acid (TCA). Collection of a 10 ml sample took about 5 s. Each flask was weighed to determine the exact volume collected. After 10 min, the pH was adjusted to between pH 6 and 8 using 10 N KOH. Standards containing 0 to 50 nmol of pyrophosphate were prepared in 10 ml of crotonate medium and treated as above. *E. coli* grown on LB medium to an absorbance of 0.6 at 600 nm was used as a positive control. Pyrophosphate was measured colorometrically as previously described ([22](#_ENREF_22)). Duplicate cultures of each organism were used and three samples were taken from each culture.

Pyrophosphatase activity was measured in both the soluble and membrane fractions. The 1 ml assay contained 20 mM TEA, 5 mM MgCl, and 1 mM pyrophosphate (pH 7.8) and was prewarmed to 37°C prior to the addition of *S. aciditrophicus* enzymes. A 50-µl sample was taken every one minute and pyrophosphate concentration was measured as above.

**AMP and ATP measurements.** *S. aciditrophicus* was grown in pure culture on 20 mM crotonate medium in triplicate 200 ml volumes. The cells were grown to mid log phase and centrifuged (8,000 x g, 10 min, 21°C) in air-tight centrifuge bottles. Cells were resuspended in 10 ml of 20 mM crotonate medium and incubated for 24 hours at 37°C. One-milliliter samples were taken before and after incubation for substrate loss determination. All procedures were performed anaerobically. After incubation, 1 ml of the culture was taken and immediately put into 500 µl of ice cold 1 M KOH and bead beat using Lysing Matrix E (MP Biomedicals) for 1 minute. Sample was centrifuged for 5 min (14,300 x g, 5 min, 4°C) and stored at -80°C. High performance liquid chromatography and a UV/VIS diode array spectrometer were used to resolve and detect AMP and ATP as described previously ([23](#_ENREF_23)). Adenylate nucleotides were separated using the following step-wise gradients of buffer A (100 mM KH_2_PO_4_ and 1.0 mM tetrabutylammonium sulfate) and buffer B (CH_3_CN): 100%/0% for 2.5 minutes, 95%/5% for 5 minutes, and 85%/15% for 7.5 minutes ([23](#_ENREF_23)). Concentrations of ATP and AMP were detected by absorption at 254 nm and quantified based on the integrated area of standards. *E. coli* strain B was grown in 200 ml minimal glucose medium aerobically as a control.  *E. coli* cultures were concentrated, and harvested as described above, with the exception of the incubation adjusted to 40 minutes after centrifugation.

**Reference**

1. **McInerney MJ, Bryant, M.P, Pfennig N.** 1979. Anaerobic bacterium that degrades fatty acids in syntrophic association with methanogens. Arch Microbiol **122**:129-135
2. **Sieber JR, Le HM, McInerney MJ**. 2014. The importance of hydrogen and formate transfer for syntrophic fatty, aromatic and alicyclic metabolism. Environ Microbiol **16**:177-188.
3. **McInerney MJ, Bryant MP, Hespell RB, Costerton JW.** 1981. *Syntrophomonas wolfei* gen. nov. sp. novel an anaerobic, syntrophic, fatty acid-oxidizing bacterium. Appl Environ Microbiol **41**:1029-1039.
4. **Atkinson DE.** 1968 The energy charge of the adenylate pool as a regulatory parameter. Interaction with feedback modifiers*.* Biochemistry. **7**: p. 4030-4.
5. **Conway T, Creecy JP, Maddox SM, Grissom JE, Conkle TL, Shadid TM, Teramoto J, San Miguel P, Shimada T, Ishihama A, Mori H, Wanner BL.** 2014. Unprecedented high-resolution view of bacterial operon architecture revealed by RNA sequencing. mBio **5**:01442-01414.
6. **Dillies MA, Rau A, Aubert J, Hennequet-Antier C, Jeanmougin M, Sevant N, Keime C, Marot G, Castel D, Estelle J, Guernec G, Jagla B, Jouneau L, Laloe D, Le Gall C, Schaeffer B, Le Crom S, Guedj M, Jaffrezic F.** 2013. A comprehensive evaluation of normalization methods for Illumina high-throughput RNA sequencing data analysis. Brief Bioinf **14**:671-683.
7. **Erde J, Loo RR, Loo JA.** 2014. Enhanced FASP (eFASP) to increase proteome coverage and sample recovery for quantitative proteomic experiments. J Proteome Res **13**:1885-1895
8. **Sibole SC, Erdemir A.** 2012. Chondrocyte deformations as a function of tibiofemoral joint loading predicted by a generalized high-throughput pipeline of multi-scale simulations. PLoS ONE **7**:e37538. doi:10.1371/journal.pone.0037538.
9. **Manza LL, Stamer SL, Ham AJ, Codreanu SG, Liebler DC.** 2005 Sample preparation and digestion for proteomic analyses using spin filters. Proteomics **5**:1742-1745.
10. **Wisniewski JR, Zielinska DF, Mann M.** 2011. Comparison of ultrafiltration units for proteomic and N-glycoproteomic analysis by the filter-aided sample preparation method. Anal Biochem **410**:307-309.
11. **Wisniewski JR, Zougman A, Nagaraj N, Mann M.** 2009. Universal sample preparation method for proteome analysis. Nat Methods **6**:359-362.
12. **Hughes MA, Silva JC, Geromanos SJ, Townsend CA.** 2006. Quantitative proteomic analysis of drug-induced changes in mycobacteria. J Proteome Res **5**:54-63.
13. **Silva JC, Denny R, Dorschel CA, Gorenstein M, Kass IJ, Li GZ, McKenna T, Nold MJ, Richardson K, Young P, Geromanos S.** 2005. Quantitative proteomic analysis by accurate mass retention time pairs. Anal Chem **77**:2187-2200.
14. **Sieber JR, Crable BR, Sheik GB, Hurst GB, Rohlin L, Gunsalus RP, McInerney MJ.** 2015. Proteomic analysis reveals metabolic and regulatory systems involved the syntrophic and axenic lifestyle of *Syntrophomonas wolfei*. Front Microbiol **6**:115. doi: 10.3389/fmicb.2015.00115.
15. **Bowman CM, Valdez RO, Nishimura JS.** 1976. Acetate kinase from *Veillonella alcalescens*. Regulation of enzyme activity by succinate and substrates. J Biol Chem **251**:3117-3121.
16. **Stadtman ER.** 1952. The purification and properties of phosphotransacetylase. *J Biol Chem* **196**:527-534.
17. **Bergmeyer HU, Holz G, Klotzsch H, & Lang G.** 1963. Phosphotransacetylase from *Clostridium kluyveri*. Culture of the bacterium, isolation, crystallization and properties of the enzyme. Biochem Z **338**:114-121.
18. **Schuhle K, Gescher J, Feil U, Paul M, Jahn M, Schagger H, Fuchs G.** 2003. Benzoate-coenzyme A ligase from *Thauera aromatica*: an enzyme acting in anaerobic and aerobic pathways. J Bacteriol **185**:4920-4929.
19. **McComb RB, Bond LW, Burnett RW, Keech RC, & Bowers GN.** 1976. Determination of the molar absorptivity of NADH. Clin Chem **22**:141-150.
20. **Szasz G, Gruber W, Bernt E.** 1976. Creatine kinase in serum: 1. Determination of optimum reaction conditions. Clin Chem **22**:650-656.
21. **Kurebayashi N, Kodama T, Ogawa Y.** 1980. P1,P5-Di(adenosine-5')pentaphosphate(Ap5A) as an inhibitor of adenylate kinase in studies of fragmented sarcoplasmic reticulum from bullfrog skeletal muscle. J Biochem **88**:871-876.
22. **Kukko E, Heinonen J.** 1982. The intracellular concentration of pyrophosphate in the batch culture of *Escherichia coli*. Eur J Biochem **127**:347-349.
23. **Lane RS, Fu Y, Matsuzaki S, Kinter M, Humphries KM, Griffin TM**. 2015. Mitochondrial respiration and redox coupling in articular chondrocytes*.* Arthritis Res Ther. **17**:54. **doi:** 10.1186/s13075-015-0566-9.
