# Supplementary material for: Pyrophosphate-Dependent ATP Formation from Acetyl Coenzyme A in Syntrophus aciditrophicus, a New Twist on ATP Formation
Source: mBio. 2016 Aug 16;7(4):e01208-16. doi: 10.1128/mBio.01208-16 (PMC4992975; doi:10.1128/mBio.01208-16)
Supplement: Figure S1 — Potential mechanisms for ATP synthesis by substrate-level phosphorylation in S. aciditrophicus. Locus tag identification number is given for each gene. Download [file mbo004162932sf1.pdf]

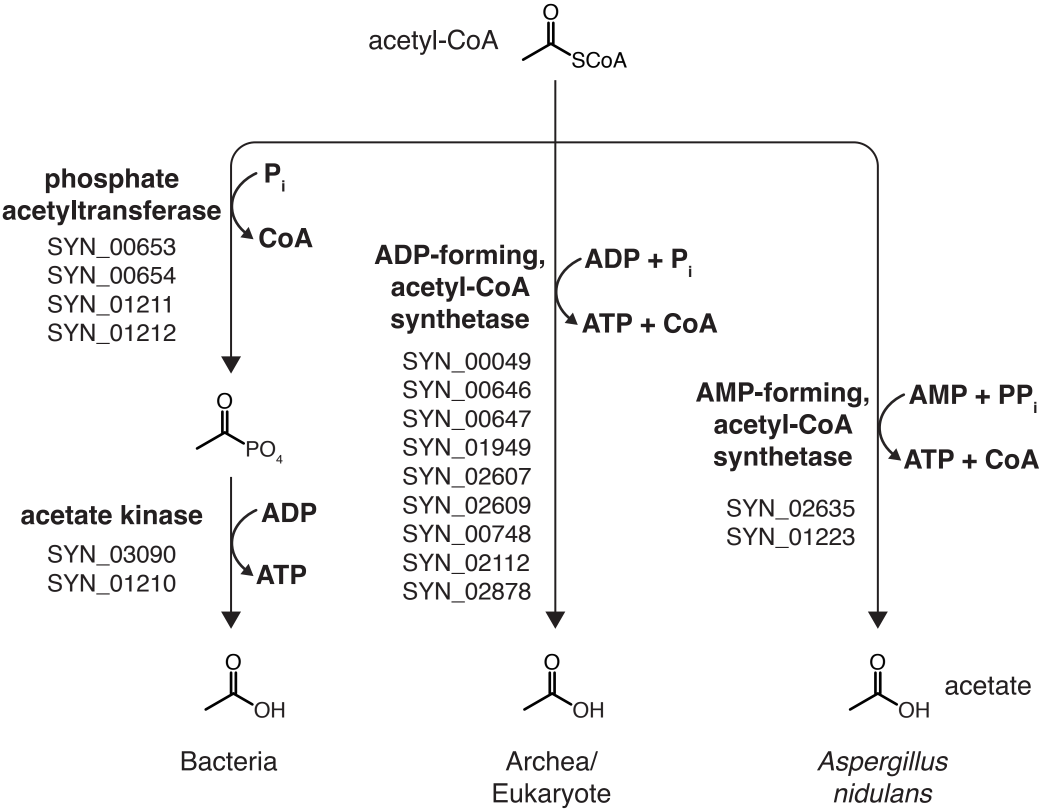

acetyl-CoA
O
SCoA
phosphate acetyltransferase
SYN_00653
SYN_00654
SYN_01211
SYN_01212
P i
CoA
O
PO4
acetate kinase
SYN_03090
SYN_01210
ADP
ATP
O
OH
Bacteria
ADP-forming, acetyl-CoA synthetase
SYN_00049
SYN_00646
SYN_00647
SYN_01949
SYN_02607
SYN_02609
SYN_00748
SYN_02112
SYN_02878
ADP + P i
ATP + CoA
O
OH
Archea/ Eukaryote
AMP-forming, acetyl-CoA synthetase
SYN_02635
SYN_01223
AMP + PP i
ATP + CoA
O
OH
acetate
Aspergillus nidulans
