# Supplementary material for: Pyrophosphate-Dependent ATP Formation from Acetyl Coenzyme A in Syntrophus aciditrophicus, a New Twist on ATP Formation
Source: mBio. 2016 Aug 16;7(4):e01208-16. doi: 10.1128/mBio.01208-16 (PMC4992975; doi:10.1128/mBio.01208-16)
Supplement: Figure S2 — Amino acid alignments of Acs1 and other representative acetyl-CoA synthetases whose biochemical function has been demonstrated. Blue denotes the known nucleotide binding sites, and green denotes the known active site amino acids. Download [file mbo004162932sf2.pdf]

```
315  GWVTGHSYVVYGPLALGATSIMFEGVPNYPTYSRFWQVVEKYKVTSFYTAPTAIRAIAKE  374  WP_011418543 Syntrophus aciditrophicus
338  GWITGHSYVVYGPLCNGATSVLFESTPVYPNAGRYWETVERLKINQFYGAPTAVRLLLKY  397  Q9NUB1 Homo sapiens
257  GWVTGTVYGIFAPWLNGATNVIVGGRF---SPESWYGTIEQLGVNVWYSAPTAFRMLMGA  313  P39062 Bacillus subtilis
308  GWVTGHSYLLYGPLACGATTLMFEGVPNWPTPARMAQVVDKHQVNILYTAPTAIRALMAE  367  P27550 Escherichia coli K12
326  GWITGHTYVVYAPLLLGCSTVVFESTPAYPDFSRYWDVIEKHKVTQFYVAPTALRLLKRA  385  P16928 Aspergillus nidulans
332  GWVTGHSYIVYAPLILGMTSLMYEGAADYPDFGRWWKNIQDHKVTVLYTAPTAVRMFMKQ  391  P27095 Methanosaeta concilii
308  GWVTGHSYLLYGPLACGATTLMFEGVPNWPTPARMCQVVDKHQVNILYTAPTAIRALMAE  367  Q8ZKF6 Salmonella typhimurium

375  GDEWLKGIDLSSLRVLSSVGEPLNPEAWQWFYEKIGNSQCPIVDTWWQTETGGHMILPLP  434  WP_011418543 Syntrophus aciditrophicus
398  GDAWVKKYDRSSLRTLGSVGEPINCEAWEWLHRVVGDSRCTLVDTWWQTETGGICIAPRP  457  Q9NUB1 Homo sapiens
314  GDEMAAKYDLTSLRHVLSVGEPLNPEVIRWGHKVFNK---RIHDTWWMTETGSQLICNYP  370  P39062 Bacillus subtilis
368  GDKAIEGTDRSSLRILGSVGEPINPEAWEWYWKKIGNEKCPVVDTWWQTETGGFMITPLP  427  P27550 Escherichia coli K12
386  GDHHIHH-KMEHLRVLGSVGEPIAAEVWKWYFEKVGKEEAHICDTYWQTETGSNVITPLA  444  P16928 Aspergillus nidulans
392  GAEWPDKYDLSSLRLLGSVGEPINPEAWMWYREHIGRGELQIMDTWWQTETGTFLNSPLP  451  P27095 Methanosaeta concilii
368  GDKAIEGTDRSSLRILGSVGEPINPEAWEWYWKKIGKEKCPVVDTWWQTETGGFMITPLP  427  Q8ZKF6 Salmonella typhimurium

435  GA--IDIKPAKPALPIMGVIPTLVDDQTGEELKNMEANGALCFKTAWPGITRTIYGDHKR  492  WP_011418543 Syntrophus aciditrophicus
458  SEEGAEILPAMAMRPFFGIVPVLMDEKGSV-VEGSNVSGALCISQAWPGMARTIYGDHQR  516  Q9NUB1 Homo sapiens
371  C---MDIKPGSMGKPIPGVEAAIVDNQGNELP--PYRMGNLAIKKGWPSMMHTIWNNPEK  425  P39062 Bacillus subtilis
428  GA--TELKAGSATRPFFGVQPALVDNEGNP-L-EGATEGSLVITDSWPGQARTLFGDHER  483  P27550 Escherichia coli K12
445  GV--TPTKPGSASLPFFGIEPAIIDPVSGEEISGNDVEGVLAFKQPWPSMARTVWGAHKR  502  P16928 Aspergillus nidulans
452  I---TPLKPGSCTFPLPGYDISILDEEGNEVP--LGSGGNIVALKPYPSMLRAFWGDKER  506  P27095 Methanosaeta concilii
428  GA--IELKAGSATRPFFGVQPALVDNEGHP-Q-EGATEGNLVITDSWPGQARTLFGDHER  483  Q8ZKF6 Salmonella typhimurium

493  YEETYFEPY------PGNYFSGDGALRDKDGYYRITGRIDDVINVSGHRMGTAEIEATLN  546  WP_011418543 Syntrophus aciditrophicus
517  FVDAYFKAY------PGYYFTGDGAYRTEGGYYQITGRMDDVINISGHRLGTAEIEDAIA  570  Q9NUB1 Homo sapiens
426  YESYFMP--------GGWYVSGDSAYMDEEGYFWFQGRVDDVIMTSGERVGPFEVESKLV  477  P39062 Bacillus subtilis
484  FEQTYFSTF------KNMYFSGDGARRDEDGYYWITGRVDDVLNVSGHRLGTAEIESALV  537  P27550 Escherichia coli K12
503  YMDTYLNVY------KGYYFTGDGAGRDHEGYYWIRGRVDDVVNVSGHRLSTAEIEAALI  556  P16928 Aspergillus nidulans
507  FMKEYWQFYWDVPGRRGVYLAGDKAQRDKDGYFFIQGRIDDVLSVAGHRIANAEVESALV  566  P27095 Methanosaeta concilii
484  FEQTYFSTF------KNMYFSGDGARRDEDGYYWITGRVDDVLNVSGHRLGTAEIESALV  537  Q8ZKF6 Salmonella typhimurium

547  AHEKVAESAVVGYPHEVKGQSIYAYITLKTGFEPSEDLKKELTQYVRTIIGPIATPEKIQ  606  WP_011418543 Syntrophus aciditrophicus
571  DHPAVPESAVIGYPHDIKGEAAFAFIVVKDSAGDSDVVVQELKSMVATKIAKYAVPDEIL  630  Q9NUB1 Homo sapiens
478  EHPAIAEAGVIGKPDPVRGEIIKAFIALREGFEPSDKLKEEIRLFVKQGLAAHAAPREIE  537  P39062 Bacillus subtilis
538  AHPKIAEAAVVGIPHNIKGQAIYAYVTLNHGEEPSPELYAEVRNWVRKEIGPLATPDVLH  597  P27550 Escherichia coli K12
557  EHPMVAEAAVVGIADELTGQAVNAFVSLKEGNETNDQVRKDLILQVRKSIGPFAAPKAVF  616  P16928 Aspergillus nidulans
567  AHPKIAEAAVVGKPDEVKGESIVAFVILRVGNEPSPELAKDAIAFVRKTLGPVAAPTEVH  626  P27095 Methanosaeta concilii
538  AHPKIAEAAVVGIPHAIKGQAIYAYVTLNHGEEPSPELYAEVRNWVRKEIGPLATPDVLH  597  Q8ZKF6 Salmonella typhimurium
```
