# Supplementary material for: Pyrophosphate-Dependent ATP Formation from Acetyl Coenzyme A in Syntrophus aciditrophicus, a New Twist on ATP Formation
Source: mBio. 2016 Aug 16;7(4):e01208-16. doi: 10.1128/mBio.01208-16 (PMC4992975; doi:10.1128/mBio.01208-16)
Supplement: Table S1 — Transcript and proteomic data of housekeeping and substrate-level phosphorylation genes and proteins, respectively, in Syntrophus aciditrophicus [file mbo004162932st1.docx]

Table S1: Transcript and proteomic data of housekeeping and substrate-level phosphorylation genes and proteins, respectively, in *Syntrophus aciditrophicus*.

| **Proteomic data**^a^ | | | | | | |
| --- | --- | --- | --- | --- | --- | --- |
| **Locus ID** | **GenBank Accession Number** | **Protein Name** | **Crotonate pure culture** | **Crotonate-coculture** | **Benzoate coculture** | **Cyclohexane carboxylate coculture** |
| SYN_01223 | WP_011418354.1 | AMP-forming, acetyl-CoA synthetase | 1,089,017 | 320,553 | 194,764 | 119,394 |
| SYN_02635 | WP_011418543.1 | AMP-forming, acetyl-CoA synthetase | 6,588,816 | 3,761,649 | 1,750,763 | 8,646,857 |
| SYN_00049 | WP_011418090.1 | ADP-forming, acetyl-CoA synthetase | ND^b^ | 222,434 | 18,239 | ND |
| SYN_00646 | WP_011417955.1 | ADP-forming, acetyl-CoA synthetase | ND | 49,693 | 9,426 | ND |
| SYN_00647 | WP_011417956.1 | ADP-forming, acetyl-CoA synthetase | ND | 100,559 | 22,258 | ND |
| SYN_00748 | WP_011418068.1 | ADP-forming, acetyl-CoA synthetase | ND | ND | ND | ND |
| SYN_01949 | WP_011416704.1 | ADP-forming, acetyl-CoA synthetase | ND | ND | ND | ND |
| SYN_02112 | WP_011416964.1 | ADP-forming, acetyl-CoA synthetase | ND | 44,771 | 46,066 | ND |
| SYN_02607 | WP_011416366.1 | ADP-forming, acetyl-CoA synthetase | ND | ND | ND | ND |
| SYN_02609 | WP_011416368.1 | ADP-forming, acetyl-CoA synthetase | ND | 119,105 | ND | ND |
| SYN_01210/  3090 | WP_011418340.1/ WP_011417964.1 | Butyrate kinase | ND | ND | 10,787 | ND |
| SYN_00653 | WP_011417962.1 | Phosphate butyryltransferase | ND | ND | ND | ND |
| SYN_00654 | WP_011417963.1 | Phosphate butyryltransferase | ND | 22,126 | 14,909 | ND |
| SYN_01211 | WP_011418341.1 | Phosphate butyryltransferase | 41,632 | 39,024 | 14,315 | ND |
| SYN_01212 | WP_011418342.1 | phosphate butyryltransferase | ND | ND | ND | ND |
| SYN_01601 | WP_011416241.1 | DNA directed RNA polymerase subunit alpha | 357,592 | 650,428 | 70,194 | 307,640 |
| SYN_02050 | WP_011415916.1 | DNA polymerase III beta chain | 172,844 | 126,432 | 52,791 | 152,331 |
| SYN_02049 | WP_011415917.1 | DNA gyrase subunit B | ND | 367,424 | 70,319 | ND |
| SYN_02048 | WP_011415918.1 | DNA gyrase subunit A | ND | 289,026 | 33,988 | ND |
|  |  | Total number of peptides detected | 207,633,196 | 298,014,665 | 107,708,548 | 262,681,056 |

| **Transcript data^a^** | | | | | |
| --- | --- | --- | --- | --- | --- |
| **Locus ID** | **GenBank Accession Number** | **Gene Name** | **Crotonate pure** | **Benzoate coculture** | **Cyclohexane carboxylate coculture** |
| SYN_01223 | WP_011418354.1 | AMP-forming, acetyl-CoA synthetase | 3.2 | 2.4 | 2.9 |
| SYN_02635 | WP_011418543.1 | AMP-forming, acetyl-CoA synthetase | 34.3 | 29.7 | 38.6 |
| SYN_00049 | WP_011418090.1 | ADP-forming, acetyl-CoA synthetase | 5.2 | 3.8 | 2.1 |
| SYN_00646 | WP_011417955.1 | ADP-forming, acetyl-CoA synthetase | 1.3 | 1.1 | 1.6 |
| SYN_00647 | WP_011417956.1 | ADP-forming, acetyl-CoA synthetase | 1.6 | 0.9 | 1.6 |
| SYN_00748 | WP_011418068.1 | ADP-forming, acetyl-CoA synthetase | 2.2 | 1.9 | 2.6 |
| SYN_01949 | WP_011416704.1 | ADP-forming, acetyl-CoA synthetase | 3.6 | 3.1 | 2.5 |
| SYN_02112 | WP_011416964.1 | ADP-forming, acetyl-CoA synthetase | 1.4 | 0.7 | 2.1 |
| SYN_02607 | WP_011416366.1 | ADP-forming, acetyl-CoA synthetase | 1.7 | 1.0 | 1.8 |
| SYN_02609 | WP_011416368.1 | ADP-forming, acetyl-CoA synthetase | 1.5 | 2.9 | 2.4 |
| SYN_01210/  3090 | WP_011418340.1/ WP_011417964.1 | Butyrate kinase | 1.7 | 0.7 | 3.9 |
| SYN_00653 | WP_011417962.1 | Phosphate butyryltransferase | 1.9 | 1.3 | 5.3 |
| SYN_00654 | WP_011417963.1 | Phosphate butyryltransferase | 2.4 | 0.9 | 5.3 |
| SYN_01211 | WP_011418341.1 | Phosphate butyryltransferase | 2.0 | 1.2 | 2.4 |
| SYN_01212 | WP_011418342.1 | phosphate butyryltransferase | 1.9 | 1.8 | 2.4 |
| SYN_01601 | WP_011416241.1 | DNA directed RNA polymerase subunit alpha | 10.0 | 24.6 | 6.0 |
| SYN_02050 | WP_011415916.1 | DNA polymerase III beta chain | 3.1 | 4.1 | 2.3 |
| SYN_02049 | WP_011415917.1 | DNA gyrase subunit B | 2.1 | 1.9 | 2.4 |
| SYN_02048 | WP_011415918.1 | DNA gyrase subunit A | 3.1 | 3.5 | 1.9 |
|  |  | Total Transcripts Detected | 48,351,774 | 20,875,168 | 718,797 |

^a^ Proteomic data are expressed as averages of the number of peptides detected for duplicate cultures normalized to the two most abundant peptides detected under the condition listed. Transcript data are expressed as the average number of transcript reads for duplicate cultures normalized to the gene length and total number of recruited transcripts to the genome for the condition listed.

^b^ND, not detected.
