# Supplementary material for: Pyrophosphate-Dependent ATP Formation from Acetyl Coenzyme A in Syntrophus aciditrophicus, a New Twist on ATP Formation
Source: mBio. 2016 Aug 16;7(4):e01208-16. doi: 10.1128/mBio.01208-16 (PMC4992975; doi:10.1128/mBio.01208-16)
Supplement: Table S2 — Purification of the acetyl-CoA synthetase activity from cell extracts of S. aciditrophicus grown on crotonate. [file mbo004162932st2.docx]

Table S2: Purification of the acetyl-CoA synthetase activity from cell-free extracts of *S. aciditrophicus* grown on crotonate.

| Purification Step | Total  Protein  (mg) | Specific Activity  (U • mg^-1^ of protein) | Total  Activity  (U) | Yield  (%) | Fold purification |
| --- | --- | --- | --- | --- | --- |
| Cell-free extract | 10.1 | 0.6 | 6.1 | 100 | 1.0 |
| 45% Ammonium sulfate soluble fraction | 10.4 | 0.4 | 4.2 | 69 | 0.7 |
| DEAE Pooled | 1.1 | 2.3 | 2.5 | 41 | 3.8 |
| Hydroxyapatite Pooled | 0.4 | 11.0 | 4.4 | 72 | 18.3 |
| Reactive Green | 0.08 | 56.9 | 4.6 | 75 | 94.8 |
