# Supplementary material for: Pyrophosphate-Dependent ATP Formation from Acetyl Coenzyme A in Syntrophus aciditrophicus, a New Twist on ATP Formation
Source: mBio. 2016 Aug 16;7(4):e01208-16. doi: 10.1128/mBio.01208-16 (PMC4992975; doi:10.1128/mBio.01208-16)
Supplement: Table S3 — Substrate specificity of Acs1 purified from cell extracts of S. aciditrophicus grown on crotonate and for the purified recombinant Acs1 (SYN_02635 gene product). [file mbo004162932st3.docx]

Table S3: Substrate specificity of Acs1 purified from cell-free extracts of *S. aciditrophicus* grown on crotonate and for the purified recombinant Acs1 (SYN_02635 gene product).

|  | Purified Acs1 | | Recombinant Acs1 | |
| --- | --- | --- | --- | --- |
| Fatty Acid | Concentration  (mM) | Specific activity^a^ | Concentration  (mM) | Specific activity |
| Acetate | 2 | 68 | 2 | 70 |
| Crotonate | 3 | 0.10 | 2 | 0.28 |
| Benzoate | 3 | 0.26 | 2 | 0.29 |
| Cyclohexane-1-carboxylate | 3 | 0.03 | 2 | 0.32 |
| Butyrate | 1 | 0.02 | 2 | 0.35 |
| Succinate | 3 | 0.42 | 2 | ND^b^ |
| Glutarate | 3 | 0.29 | 2 | ND |
| Cyclohexene -1-carboxylate | 3 | 0.22 | 2 | 0.38 |

^a^ Activities in U • mg^-1^ of protein of duplicate determinations.

^b^ ND, not determined.
