# Supplementary material for: Pyrophosphate-Dependent ATP Formation from Acetyl Coenzyme A in Syntrophus aciditrophicus, a New Twist on ATP Formation
Source: mBio. 2016 Aug 16;7(4):e01208-16. doi: 10.1128/mBio.01208-16 (PMC4992975; doi:10.1128/mBio.01208-16)
Supplement: Table S4 — Members of the Clostridia that have a gene for an AMP-forming, acetyl-CoA synthetase (ACS) and lack either one or all of the following: acetate kinase (AK), butyrate kinase (BK), phosphate acetyltransacetylase (PTA), and phosphate butyryltransferase (PTB). [file mbo004162932st4.docx]

Table S4: Members of the Clostridia that have a gene for an AMP-forming, acetyl-CoA synthetase (ACS), and lack either one or both acetate kinase (AK), butyrate kinase (BK), phosphate acetyltransacetylase (PTA), and phosphate butyryltransferase (PTB).

| Organism | Genome Identification Number for NCBI^a^ | Number of genes in the genome | | | | |
| --- | --- | --- | --- | --- | --- | --- |
|  |  | ACS | AK | PTA | BK | PTB |
| *Ammonifex degensii* (strain DSM 10501 / KC4) | 260891930 | 2 | 0 | 0 | 0 | 0 |
| *Heliobacterium modesticaldum* (strain ATCC 51547 / Ice1) | 255961476 | 2 | 1 | 0 | 0 | 0 |
| *Moorella thermoacetica* (strain ATCC 39073) orginally isolated as *Clostridium thermoaceticum* | 83588874 | 1 | 1 | 0 | 0 | 0 |

^a^All BLAST searches were performed on HAMAP (http://hamap.expasy.org/) on 01/16/2014.
